# Pauson–Khand reaction of fluorinated compounds

**DOI:** 10.3762/bjoc.16.138

**Published:** 2020-07-14

**Authors:** Jorge Escorihuela, Daniel M Sedgwick, Alberto Llobat, Mercedes Medio-Simón, Pablo Barrio, Santos Fustero

**Affiliations:** 1 Departamento de Química Orgánica, Facultad de Farmacia, Universitat de València, Av. Vicent Andrés Estellés s/n, 46100 Burjassot, Valencia, Spain,; 2 Departmento de Química Orgánica e Inorgánica, Facultad de Química, Universidad de Oviedo, Av. Julián Clavería 8, Campus Universitario de El Cristo, 33006 Oviedo, Spain

**Keywords:** alkene, alkyne, enyne, fluorine, Pauson–Khand

## Abstract

The Pauson–Khand reaction (PKR) is one of the key methods for the construction of cyclopentenone derivatives, which can in turn undergo diverse chemical transformations to yield more complex biologically active molecules. Despite the increasing availability of fluorinated building blocks and methodologies to incorporate fluorine in compounds with biological interest, there have been few significant advances focused on the fluoro-Pauson–Khand reaction, both in the inter- and intramolecular versions. Furthermore, the use of vinyl fluorides as olefinic counterparts had been completely overlooked. In this review, we collect the advances both on the stoichiometric and catalytic intermolecular and intramolecular fluoro-Pauson–Khand reaction, with special attention to the PKR of enynes containing a fluoride moiety.

## Introduction

The prevalence of fluorine-containing molecules in drug-discovery programs is nowadays unquestionable [1–3]. The presence of fluorine atoms or fluorine-containing units at strategic positions in a drug candidate may result in not only an increase in its potency, but also, perhaps more importantly, bring about an enhanced pharmacokinetic profile resulting in a more “drug-like” molecule [4]. Subtle changes in physicochemical properties such as acidity/basicity, lipophilicity, preferred conformation or hydrogen bond forming ability, among others, may result in a dramatic effect in the therapeutic potential of a drug candidate [5–6]. All these properties may be fine-tuned by the selective incorporation of fluorine into the structure of the molecule [7]. In addition to therapeutic use, fluorine-containing molecules have also found key applications in the field of medical diagnosis. Two of the most powerful imaging techniques used nowadays, positron-emission tomography (PET) and magnetic resonance imaging (MRI), are routinely carried out using fluorine-containing organic compounds [8–9]. The former takes advantage of the superb properties of the ^18^F-radioisotope as positron emitter (*t*_1/2_ = 109 min, 97% β*^+^* emission), while the latter benefits from the excellent performance of the ^19^F isotope in NMR (100% natural abundance, high sensitivity, lack of endogenous background signal). In addition, the use of fluorinated organic compounds in other key industrial fields is also of paramount importance. The agrochemical and materials industries, together with the aforementioned pharmaceutical industry, are perhaps the fields where organofluorinated compounds have exerted the most profound influence [10–12].

Regarding the development of new synthetic methodologies for the preparation of such molecules, these can be divided in two main categories: fluorination reactions [13–16] and the use of fluorinated building blocks [17]. The difference between them is that while in the former a fluorine atom or a fluorinated unit (e.g., a CF_3_ group) is introduced into a non-fluorinated molecule, the latter takes advantage of a substrate that already contains fluorine in order to achieve more complex fluorinated structures. According to these definitions, the chemistry that will be discussed in this review belongs to the fluorinated building blocks category. More specifically, the participation of fluorine-containing olefins and alkynes in the Pauson–Khand cyclization, with a special focus on the intramolecular version using fluorinated enynes, which will be discussed in detail.

The focus of this review is to highlight the efforts made in the field of the Pauson–Khand reaction with fluorinated compounds for the preparation of bicyclic derivatives.

## Review

### The Pauson–Khand reaction

**Scheme 1:**
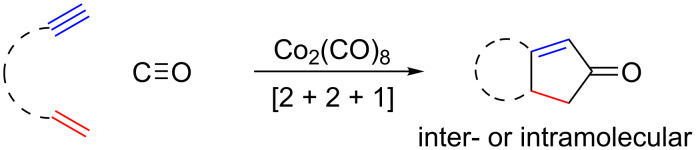
Schematic representation of the Pauson–Khand reaction.

The Pauson–Khand reaction (PKR) formally consists of a [2 + 2 + 1]-cycloaddition between an alkyne, an olefin and carbon monoxide, resulting in the regioselective formation of a cyclopentenone derivative (Scheme 1) [18–22]. This cobalt-mediated reaction was initially discovered by Pauson and Khand in the early 70s [23–25] and has since become a powerful transformation widely used in the synthesis of polycyclic complex molecules. The intermolecular variant shows a wide alkyne scope, but in terms of the olefin counterpart is limited to the use of ethylene or strained alkenes, such as norbornene and norbornadiene. The high prevalence of five-membered ring systems in natural products, pharmaceuticals and other added-value compounds accounts for the great applicability that this reaction has found [26–32]. Despite the increasing demand of fluorinated compounds and the impressive development of the PKR, the combination of these two fields has been understudied, making it an exciting field of research.

**Scheme 2:**
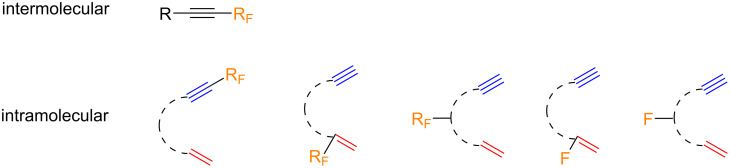
Substrates included in this review.

In order to study the influence on the reaction outcome, a fluorine atom or fluorine-containing group can be installed at either unsaturated counterpart, bound to either the olefin and/or the alkyne (vide infra) (Scheme 2). Of course, in the intramolecular version, the fluorine atom or fluorinated group can also form a part of the linker. The reaction yields are dependent on the degree of substitution, bulkiness, and electronic effects of the substituents of both the alkyne and alkene moieties. In general, electron-deficient alkynes are poor substrates for the PKR as they are deactivated in the cobalt-complexation step, and the highest yields are usually obtained with terminal alkynes. The scenario is similar in the case of fluorinated substrates, with the intramolecular version being much more developed than the intermolecular one.

**Scheme 3:**
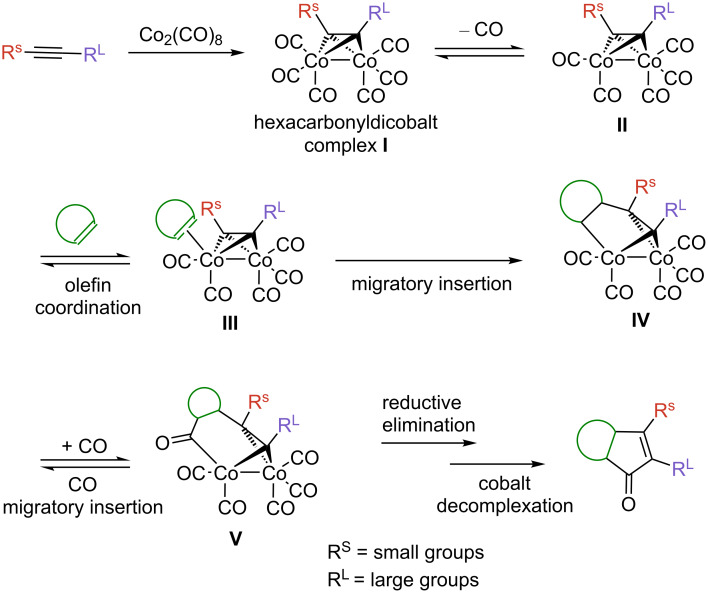
Commonly accepted mechanism for the Pauson–Khand reaction.

The regio- and stereochemistry of this transformation is predictable, in most cases. Since this is an important issue throughout the review, a concise remark about the mechanism of this transformation is outlined below (Scheme 3). This mechanism was proposed by Magnus over 30 years ago and is still valid nowadays [33], although only the first intermediate (**I**) has been isolated and characterized [34]. However, contributions by Nakamura, Pericàs, and others support the initial proposal both experimentally and theoretically [35–36]. Firstly, two coordination vacancies are freed after the extrusion of two carbon monoxide ligands from the starting cobalt species, allowing the alkyne group to bind to the cobalt metal centers. The subsequent coordination of the olefin counterpart requires the extrusion of a third carbon monoxide ligand, leading to pentacarbonyl complex **II**. This highly endotermic process is the rate-limiting step and long reaction times are generally associated to this. However, the reaction can be accelerated in conditions that facilitate the dissociation of CO ligands such as heating, microwave irradiation [37–38], visible light, or ultrasonication [39]. Alternatively, mild oxidizing additives such as amine oxides, aminophosphines, phosphine oxides, and sulfoxides may be used as promoters to facilitate the dissociation step, by oxidatively removing one of the CO ligands in form of CO_2_ [40]. The most common oxidants are *N*-morpholine *N*-oxide (NMO), trimethylamine *N*-oxide (TMANO), and dimethyl sulfoxide (DMSO). Once a new coordination vacancy has been opened on one of the cobalt centers, coordination of the olefin sets the stage for the subsequent C–C bond forming steps. The olefin is inserted into the less hindered Co–C bond, determining both the regio- and stereochemical outcome of the overall process. A carbon monoxide ligand then undergoes migratory insertion into one of the Co–C bonds in cobaltacycle **V**, followed by reductive elimination to release the final product (Scheme 3).

**Scheme 4:**
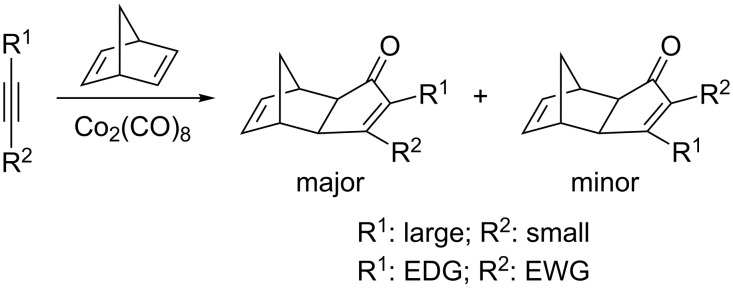
Regioselectivity of the PKR.

As mentioned above, the regiochemistry of this transformation is, in most cases, predictable for unsymmetrical alkynes (things are more complex regarding the olefin). Generally, the most sterically encumbered substituent of the alkyne occupies the proximal position in the enone system. This is dictated by the migratory insertion of the olefin into the most accessible Co–C bond (Scheme 3). This trend is strictly followed by terminal alkynes, for which exclusive formation of the α-substituted enone is observed, while mixtures are usually obtained with internal dissymmetric alkynes, although the major product follows the aforementioned trend [41]. On the other hand, for alkynes bearing groups of comparable steric demand at both ends of the alkyne, electronic effects come into play. Here, the more electron-rich substituent occupies the α position, and the more electron-poor the β position (Scheme 4). This selectivity could be rationalized by the insertion of the olefin into the weakest Co–C bond. These electronic effects have been shown to be less important than steric ones, and are often overcome by the latter. Regarding the stereochemistry, *exo*-products are almost exclusively obtained for norbornene and norbornadiene.

Many deviations from the classic reaction conditions have been described, including the use of metals other than cobalt (such as rhodium, iridium, titanium, ruthenium, nickel, and palladium), or the use of CO surrogates such as aldehydes, alcohols and formates. Recently, its utility in flow chemistry has also been described [42].

### Intramolecular Pauson–Khand reactions of fluorine-containing compounds

**Scheme 5:**
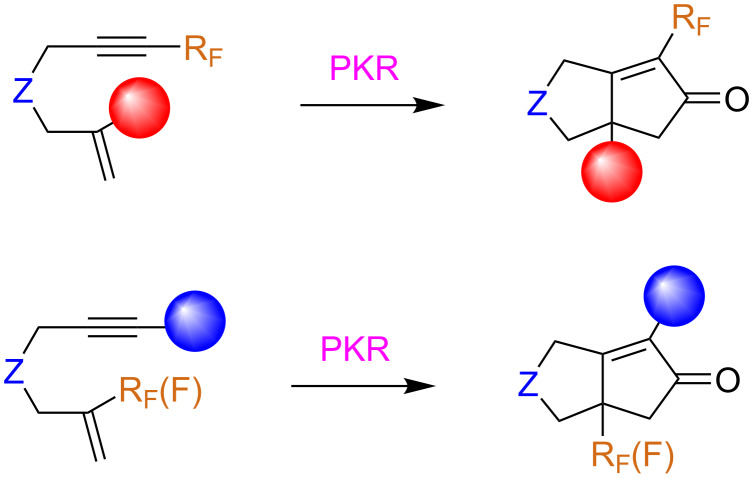
Variability at the acetylenic and olefinic counterpart.

The utility of the Pauson–Khand reaction in the preparation of polycyclic compounds bearing both nitrogenated and cyclopentenone rings, two ubiquitous domains in drugs and natural products, has been reported in various contributions using 1,*n*-enynes, particularly 4-aza-1,7-enynes as starting materials [43–45]. However, the synthesis of fluorinated 1,*n*-enynes as well as the corresponding Pauson–Khand adducts has, until recently, scarcely been described in the literature. The intramolecular version of this reaction has recently gained recognition since it facilitates the synthesis of cyclopentenone-fused ring systems, which tend to be difficult to construct. The Pauson–Khand reaction has also been used as a key step in the synthesis of a number of biologically-relevant compounds, including fluorine-containing piperidine-fused cycles. Of course, where the fluorinated group is positioned in the final compound depends on whether it is attached to the alkene or alkyne counterpart of the substrate (Scheme 5).

**Scheme 6:**
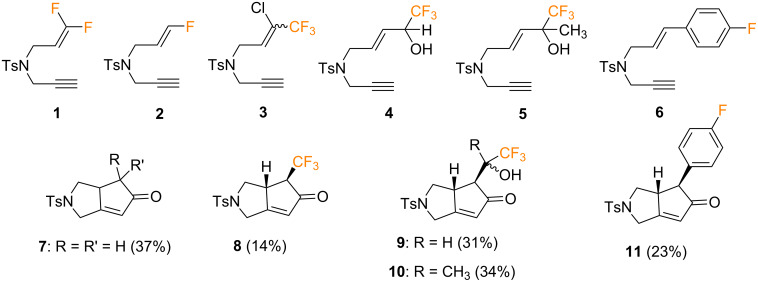
Pauson–Khand reaction of fluoroolefinic enynes reported by the group of Ishizaki [46].

Regarding the intramolecular PKR of fluorinated enynes, only a few examples have been described. The first example was reported in 2001 by Ishizaki and co-workers [46]. In this study, a wide variety of 1,6-enynes bearing fluorine atoms or fluorine-containing groups at the alkenyl or alkynyl positions were synthesized and evaluated as substrates in the intramolecular PKR with dicobalt octacarbonyl [Co_2_(CO)_8_] in CH_2_Cl_2_ and promoted by NMO. In general, the presence of fluorinated groups on the alkenyl moiety of the 1,6-enyne resulted in low yields (lower than 35%) of the corresponding cyclized products, due to the poor reactivity of the fluorinated olefin (Scheme 6). For example, difluoroalkene-containing compound **1** decomposed and no cyclized product was formed. In this NMO-promoted PKR, monofluoroolefinic enyne **2** afforded the defluorinated cyclopentenone **7** in 37% yield. Similarly, trifluoromethyl-substituted olefin **3** also lost the chlorine atom upon cyclization to give **8** as a single diastereoisomer, albeit in a low 14% yield. The reaction of trifluoromethyl-substituted allylic alcohols **4** and **5** afforded the corresponding cyclized products **9** and **10** (31% and 34% yield, respectively) as inseparable mixtures of diastereoisomers. Finally, 1,6-enyne **6**, bearing a 4-fluorophenyl group on the olefin, stereoselectively produced *trans*-oriented arylcyclopentenone **11** in 23% yield.

**Scheme 7:**
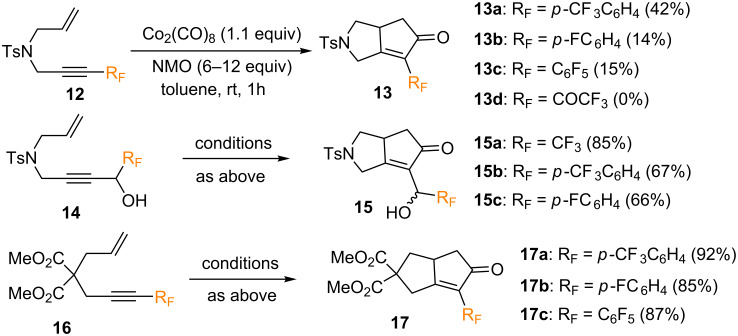
PKR of enynes bearing fluorinated groups on the alkynyl moiety, reported by the group of Ishizaki [46]. Reaction conditions: i) Co(CO)_8_ (1.1 equiv), toluene, rt, 1 h; ii) NMO (6–12 equiv), rt, 1 h.

When investigating the intramolecular PKR of enynes bearing fluorine groups on alkynyl moiety (**12**, **14**, **16**), several trends could be observed (Scheme 7). Firstly, fluoroaromatic enynes **12a**–**c** afforded the corresponding cyclized products **13a**–**c** in low yields (14–42%). However, no cyclized product was observed when using the trifluoromethyl ketone derivative **12d**. Secondly, PKR with enynes **14** containing fluorinated propargyl alcohol groups yielded diastereoisomeric mixtures of pyrrolidine ring-fused cyclopentenones **15** in good yields (67–85%) but low diastereoselectivities. Finally, the reaction of dimethyl malonate-derived fluoroaromatic enynes **16** afforded the corresponding cyclopentenone products **17** in higher yields (85–92%). Thus, the reaction of enynes bearing fluorinated groups attached to the alkyne moiety was found to afford the corresponding cyclized products in moderate to high yields, except for those bearing marked electron withdrawing groups (Scheme 7).

**Scheme 8:**
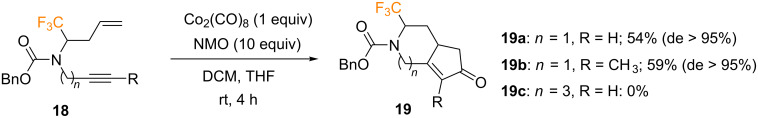
Intramolecular PKR of 1,7-enynes reported by the group of Billard [47].

In 2005, Billard and co-workers reported the PKR of α-trifluoromethylated homoallylamine derivatives (Scheme 8) [47]. Both 1,7-enynes **18a** and **18b** (*n* = 1) underwent PKR in the presence of 1 equiv of Co_2_(CO)_8_ and 10 equiv of NMO, yielding bicyclic derivatives **19** in moderate yields and high diastereoselectivity (de > 95%). The observed diastereoselectivity was rationalized considering two transition states of the PKR, and assuming the CF_3_ group occupies an axial position due to the steric and electrostatic repulsions that occur in the equatorial position. Consequently, in the most favorable transition state there is no steric hindrance between the CF_3_ group and the ethylenic hydrogen, leading to the observed diastereoisomer. On the other hand, the use of 1,9-enyne **18c** (*n* = 3) did not afford the corresponding bicyclic compound since the double and triple bonds are too distant.

**Scheme 9:**
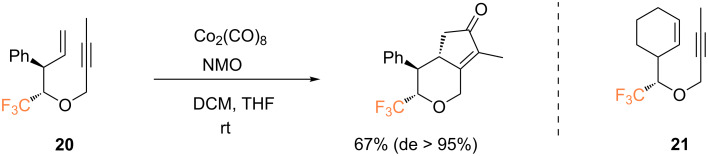
Intramolecular PKR of 1,7-enynes reported by the group of Billard [48].

In a following paper by Billard and co-workers, the PKR of oxygen-containing 1,7-enynes was assayed, affording trifluoromethylated oxygenated bicyclic enones (Scheme 9) [48]. Under classical stoichiometric conditions (reaction with Co_2_(CO)_8_ followed by the addition of NMO), and starting from the pure *anti* diastereoisomer of 1,7-enyne **20**, the expected bicyclic enone was obtained in good yield and high diastereoselectivity (de > 95%). An attempt to extend the PKR to the formation of a fused tricyclic structure, starting from 1,7-enyne **21***,* was unsuccessful and no tricyclic product was formed.

**Scheme 10:**
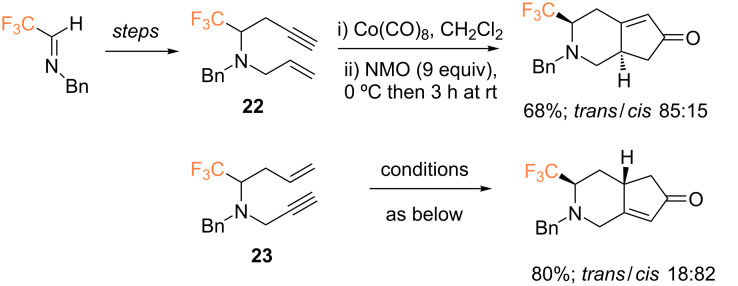
Intramolecular PKR of 1,7-enynes by the group of Bonnet-Delpon [49]. Reaction conditions: i) Co(CO)_8_ (1.2 equiv), CH_2_Cl_2_, 30 min; ii) NMO (9 equiv), 0 °C then 3 h at rt.

Bonnet-Delpon and co-workers reported the one-pot synthesis of several CF_3_-containing *N-*tethered amines in good yields (54–86% over 2 steps) [49]. These products were subjected to metathesis reactions in the presence of Grubbs catalyst affording the corresponding CF_3_-containing dehydropiperidine derivatives in excellent yields. Additionally, enynes **22** and **23** were evaluated as substrates in the intramolecular PKR, yielding the corresponding CF_3_-containing heterobicyclic derivatives in 68% (85:15 ratio of *trans*/*cis* stereoisomers) and 80% yield (18:82 ratio of *trans/cis* stereoisomers), respectively (Scheme 10).

**Scheme 11:**
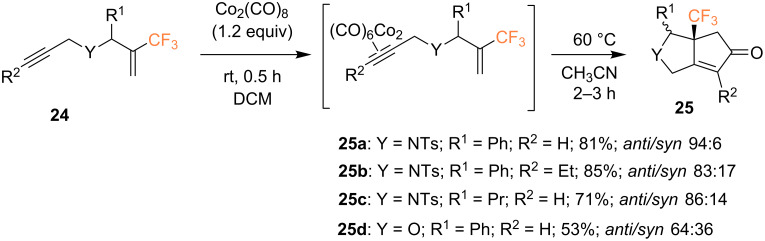
Intramolecular PKR of 1,6-enynes reported by the group of Ichikawa [50].

Ichikawa and co-workers described an attractive route to synthesize pyrrolidine ring-fused fluorinated cyclopentenone analogs via intramolecular PKR starting from 2-bromo-3,3,3-trifluoroprop-1-ene [50–51]. To this end, *N*-propargyl-*N*-[2-(trifluoromethyl)allyl]amides **24** were treated with dicobalt octacarbonyl to afford the cobalt alkyne complex, which was then heated in CH_3_CN. Under these conditions, trifluoromethylated cyclopentenone **25a** was obtained in high yield (81%) and diastereoselectivity (*anti*/*syn* = 94:6) (Scheme 11). The cyclization of internal alkyne substrate **24b** yielded pyrrolidine ring-fused cyclopentenone **25b** in similar yield but lower diastereoselectivity. Finally, *N*-propargyl-*N*-[2-(trifluoromethyl)allyl] ether **24d**, containing an ether linkage instead of the aforementioned sulfonamide linkage, gave furan ring-fused cyclopentenone **25d** in both lower yield (53%) and lower diastereoselectivity.

**Scheme 12:**
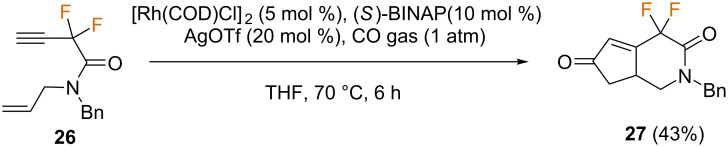
Intramolecular Rh(I)-catalyzed PKR reported by the group of Hammond [52].

A catalytic PKR of fluorinated 1,7-enyne amides **26** using catalytic amounts of [Rh(COD)Cl]_2_ was reported in 2008 by Hammond and co-workers [52]. The authors concluded that the reaction was highly sensitive to experimental parameters such as solvent, concentration, temperature, catalyst and silver salt. Under standard reaction conditions, no reaction was observed in the absence of a silver salt, and the best results were obtained in the presence of AgOTf (20 mol %), giving the corresponding *gem*-difluorinated bicyclic lactam **27** in 43% yield (Scheme 12). This reaction was limited to unsubstituted alkynes, as the PKR did not occur with a phenyl-substituted alkyne. Unfortunately, no asymmetric induction was observed.

**Scheme 13:**
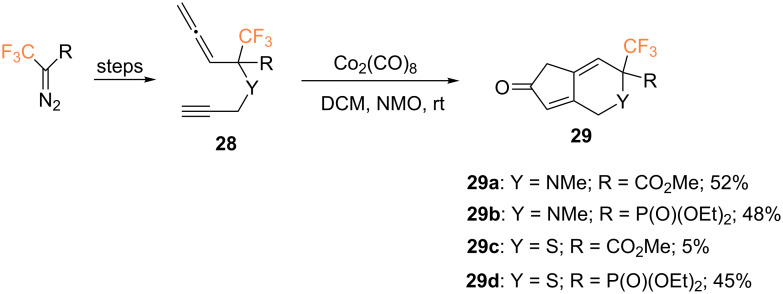
Intramolecular PKR of allenynes reported by the group of Osipov [53].

In 2011, Osipov and co-workers investigated the cobalt-mediated PKR of allenynes **28** in order to synthesize trifluoromethylated nitrogen- and sulfur-based bicyclic compounds (Scheme 13) [53]. Using this methodology, the corresponding cyclopentenones **29** were isolated in generally good yields, except for sulfur-containing derivative **29c**, due to the oxidation of the sulfide under the reaction conditions. In contrast, the phosphorus analog **29d** was obtained in 45% yield, which could be explained by favorable electronic and steric effects of the phosphonate group.

**Scheme 14:**
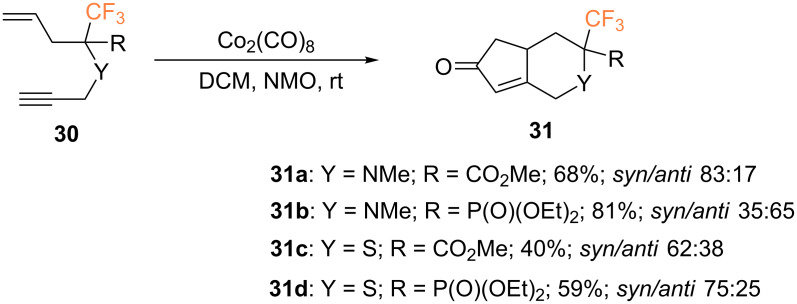
Intramolecular PKR of 1,7-enynes reported by the group of Osipov [53].

In the same work, the authors also evaluated the PKR of CF_3_-substituted enynes **30**. In this case, bicyclic products **31** were formed as mixtures of separable diastereoisomers, which could be isolated in higher yields than the products of the corresponding reaction with allenynes (Scheme 14).

**Scheme 15:**
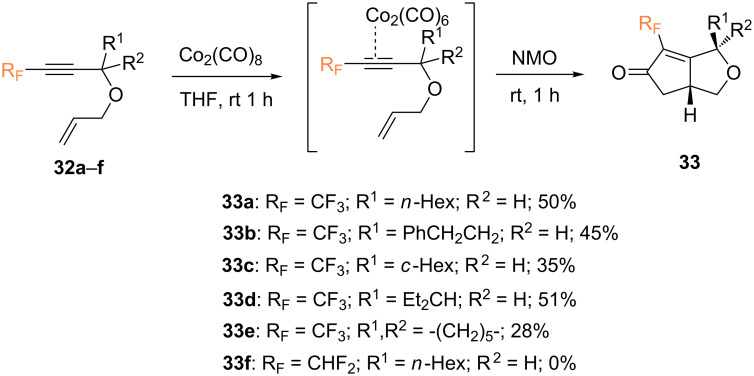
Intramolecular PKR of fluorine-containing 1,6-enynes reported by the Konno group [54].

In 2012, Konno and co-workers studied the intramolecular PKR using fluorine-containing 1,6-enynes **32** (Scheme 15) [54]. The PKR of fluorinated propargyl allyl ether **32a** afforded the corresponding *cis* bicyclic product **33a** in moderate yield and high diastereoselectivity (dr > 20:1). Other fluorinated allyl propargyl ethers **32b**–**d** afforded the corresponding bicyclic PK-adducts **33** in moderate chemical yields but with high *cis*-selectivity. On the other hand, spirocyclic derivative **33e** was formed in a significantly lower yield. Surprisingly, a subtle change of the fluoroalkyl group from a CF_3_ group to a CHF_2_ group completely inhibited the reaction.

**Scheme 16:**
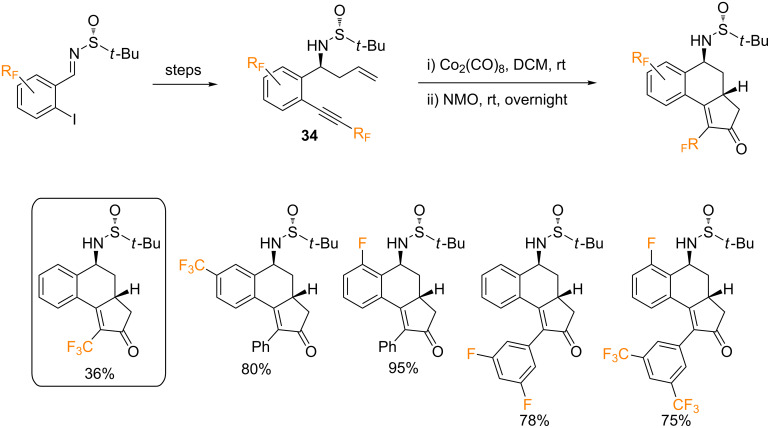
Diastereoselective PKR with enantioenriched fluorinated enynes **34** [55].

Within the frame of a broader study, our group reported a single example of an intramolecular PKR using an Ellman’s imine-derived CF_3_-containing enyne, bearing the trifluoromethylethynyl group at the *ortho* position (Scheme 16) [55]. One of the key steps in the preparation of the starting 1,*n*-enynes was a highly diastereoselective allylation reaction of chiral Ellman’s sulfinylimines. Based on this strategy, chiral 1,7-enynes **34** were prepared in three steps from sulfinylimines derived of *o*-iodobenzaldehydes. A variety of fluorinated compounds bearing fluorine or fluoroalkyl groups attached to the aryl moieties were efficiently prepared (Scheme 15). In this report, the suitability of enynes bearing CF_3_-substituted alkyne moieties (R = CF_3_) to participate in intramolecular PK reactions was also demonstrated. Furthermore, several other substrates bearing fluorine at different positions were included (Scheme 16). The process took place with moderate to high chemical yields and diastereoselectivities. This transformation can be performed on a multigram scale, and is characterized by a broad substrate scope, functional group compatibility, and high chemo- and diastereoselectivity.

**Scheme 17:**
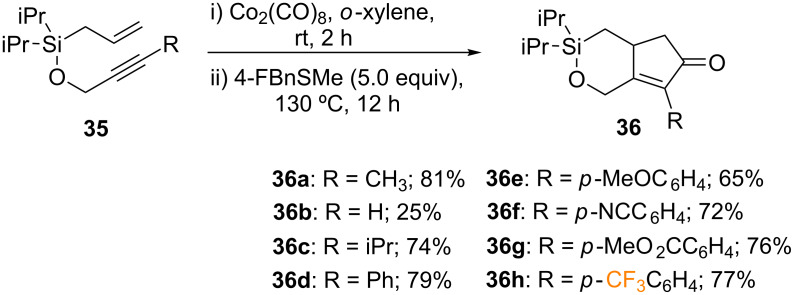
Intramolecular PKR reported by the group of Martinez-Solorio [56].

Martínez-Solorio and co-workers reported an intramolecular PKR of Si−O tethered 1,7-enynes **35**, affording cyclopentaoxasilinones **36** with high diastereoselectivity [56]. In contrast to previous silicon-based tethers, which reacted in low yields and resulted in unexpected byproducts, this transformation could be performed on a multigram scale and showed a wide substrate scope and functional group compatibility, as well as high diastereoselectivity [57–58]. In this work, Si−O tethered 1,7-enynes underwent the PKR after treatment with 1.05 equiv of Co_2_(CO)_8_ using 4-fluorobenzyl(methyl)sulfide (4-FBnSMe) as an additive, which is commercially available and can be easily recovered by flash chromatography. Under the aforementioned conditions, cyclopentaoxasilinone **36a** was isolated in 81% yield. A systematic study of the scope showed that unsubstituted enyne **35b** only afforded the desired product in 25% yield. In contrast, isopropyl and phenyl substituted enynes yielded cyclopentaoxasilinone **36c**,**d** in 74 and 79% yield, respectively. Furthermore, electron-withdrawing *para*-substituted arenes were obtained in good yields and demonstrate excellent functional group compatibility; MeO– (**36e**, 65%), −CN (**36f**, 72%), −CO_2_Me (**36g**, 76%) and fluorinated groups such as *p*-CF_3_C_6_H_4_ (**36h**, 77%) (Scheme 17).

**Scheme 18:**
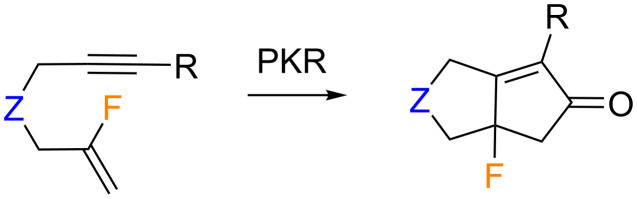
Fluorine substitution at the olefinic counterpart.

Concerning the intramolecular PKR with fluorine atoms or fluorinated groups at the vinylic position, very few examples have been described to date. In this context, our group recently explored the reactivity of 1,*n*-enynes bearing a vinyl fluoride moiety as the olefin counterpart in the intramolecular PKR [59] (Scheme 18).

**Scheme 19:**
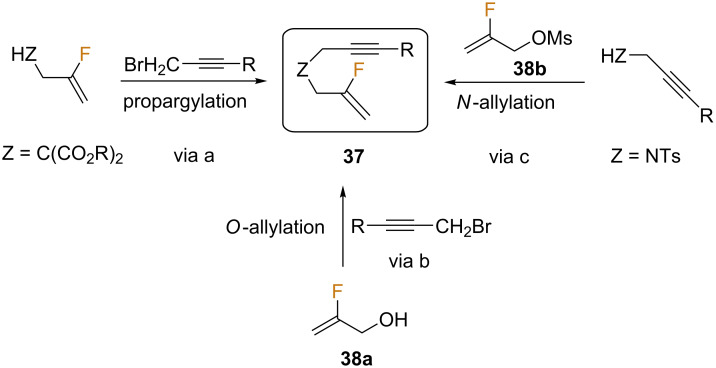
Synthesis of fluorinated enynes **37** [59].

The study of the behavior of this kind of compounds in the PK reaction started with fluorinated enynes derived from malonates and those containing heteroatoms as linkers. The synthesis of the starting enynes **37** was accomplished following various approaches (Scheme 19).

The first of these (via a) was based on a report by Hammond and co-workers, in which they detailed the Markovnikov hydrofluorination of alkynes using HF^.^DMPU coupled with a gold catalyst [60]. Accordingly, the appropriate propargylmalonate derivatives were fluorinated to give fluoroalkene intermediates, which were then converted into malonate-based enynes **37** (Z = CO_2_R) through a simple propargylation procedure. In addition, the fluoroallyl alcohol **38a** was employed as a starting material to obtain the corresponding propargyl ethers **37** (Z = O) by Williamson’s synthesis using propargyl bromides in moderate yields (via b). Activation of the fluoroallyl alcohol by conversion into the corresponding mesylate **38b** proved sufficient for the synthesis of *N*-tethered substrates **37** (Z = NTs), through simple nucleophilic substitution (via c).

**Scheme 20:**
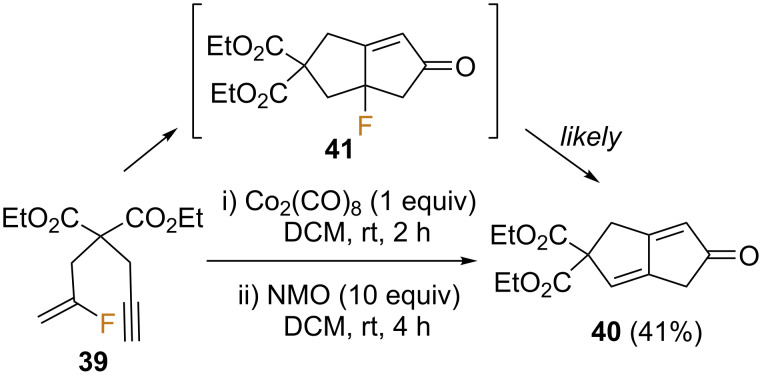
Fluorine-containing substrates in PKR [59].

It is noteworthy that under standard PK reaction conditions, fluoroenyne **39** evolves to diene **40**. The formation of compound **40** is possible by elimination of HF from the PK product **41**, meaning that the fluoro-PKR does initially take place and that the desired product **41** could be isolated by avoiding the subsequent elimination reaction. This hypothesis was confirmed when the less basic DMSO was used as the promoter instead of NMO, allowing the successful isolation of **41** (Scheme 20) [59].

**Scheme 21:**
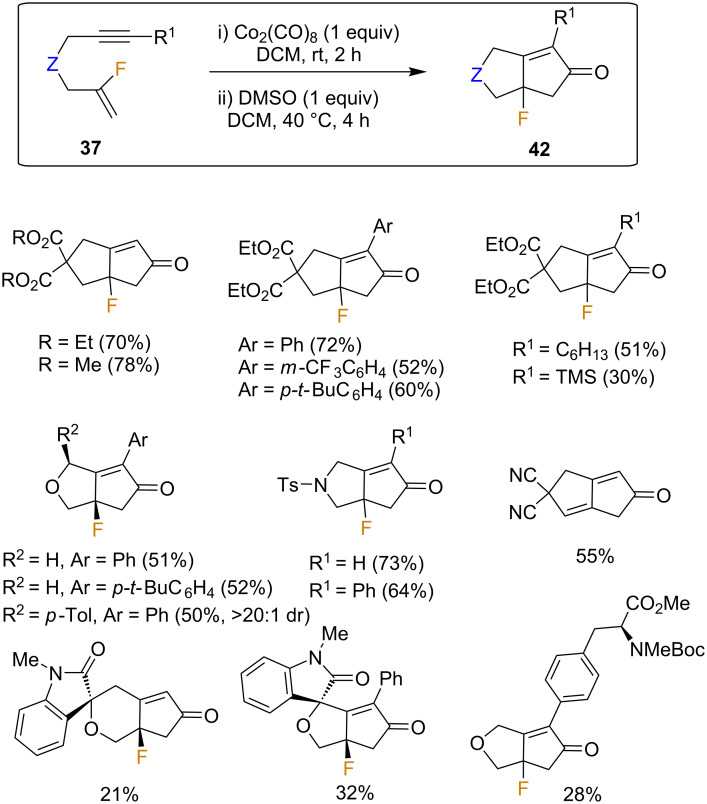
Pauson Khand reaction for fluorinated enynes by the Fustero group: scope and limitations [59].

Under the optimized conditions, using stoichiometric Co_2_(CO)_8_ and DMSO as the promoter, the process worked well and moderate to good yields were obtained for derivatives **42** bearing aryl substituents at the propargyl moiety, regardless of their electronic nature (Scheme 21). Alkyl-substituted derivatives proved to be similarly successful substrates; however, the TMS-substituted derivative was obtained in a significantly lower yield. In terms of linkers, heteroatoms were well-tolerated, although the use of malononitrile resulted exclusively in the elimination product despite testing a variety of reaction conditions. More complex biorelevant examples such as isatin derivatives were also suitable substrates, affording the corresponding spirocyclic derivatives, albeit in low yields (Scheme 21) [59]. Unfortunately, thioether and sulfone-based linkers were unsuitable in this reaction, and the starting materials were recovered in all cases.

**Scheme 22:**
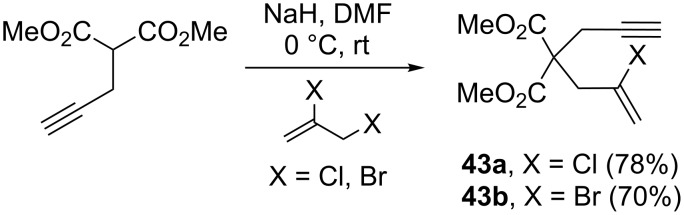
Synthesis of chloro and bromo analogues [59].

As a comparison, the same authors also explored the reactivity of the corresponding chloro- and bromoenynes **43** as olefinic counterparts for the intramolecular PKR (Scheme 22) [59].

**Scheme 23:**
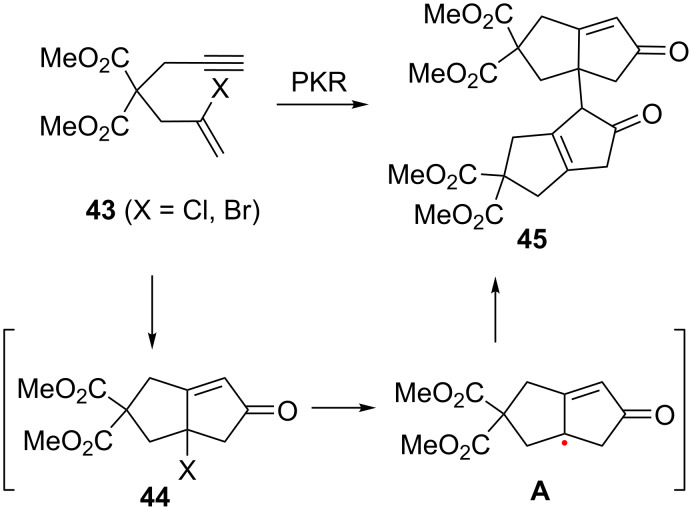
Dimerization pathway [59].

In these cases, the corresponding halogenated PKR adducts **44** could not be detected in the crude reaction mixtures. Instead, the major isolated species was dimer **45**. However, the fact that **45** bears the cyclopentenone core suggests that the desired PKR does indeed take place, albeit as an intermediate before a secondary transformation to form the final dimer (Scheme 23) [59]. The authors rationalized the formation of **45** by considering that the inherent weakening of the C—X bond going down the halogen series may favor the generation of radical **A** with chloride and bromide derivatives, especially given the tertiary position of the halide and the stoichiometric quantities of cobalt present in the reaction mixture.

**Scheme 24:**
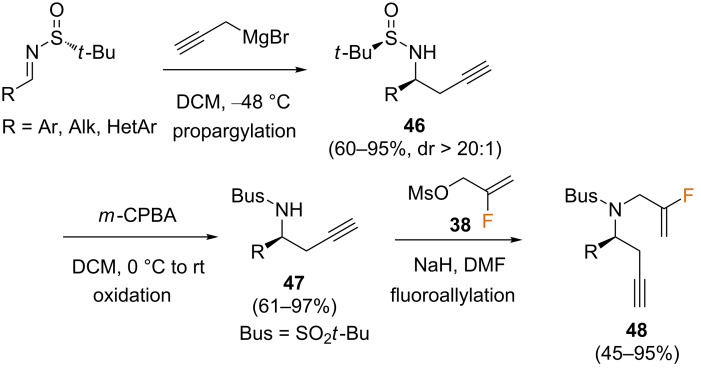
Synthesis of fluorine-containing *N*-tethered 1,7-enynes [61].

The same group also studied the intramolecular PKR of chiral fluorine-containing *N*-tethered 1,7-enynes **48** for the stereoselective construction of enantioenriched bicyclic alkaloid analogues **49**, containing a fluorine atom in the bridge position [61]. For this purpose, 1,7-enynes **48** were prepared from Ellman’s *tert*-butane sulfinylimines, followed by a diastereoselective addition of propargylmagnesium bromide to obtain a variety of sulfinyl amide intermediates **46** in good yields and high diastereoselectivities. Subsequent oxidation to the corresponding sulfonamides **47**, followed by the introduction of the fluoroallyl group via *N-*alkylation with previously described mesylate **38** provided fluorinated enynes **48** (Scheme 24).

**Scheme 25:**
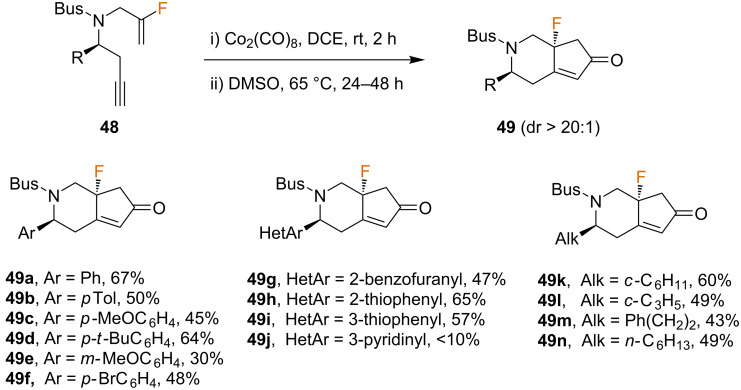
Intramolecular PKR of chiral *N*-tethered fluorinated 1,7-enynes [61].

The enantioenriched starting materials **48** were then evaluated in the Co-mediated PKR to yield the bicyclic products **49** in moderate to good yields and excellent diastereoselectivities (dr > 20:1) (Scheme 25). The substrate scope revealed that the reaction was tolerant of a wide range of substituents at the stereogenic center, such as heteroaromatic, aromatic, and aliphatic substituents (both linear and cyclic). Regarding aromatic substituents, electron-neutral and electron-rich rings with several substitution patterns performed well. However, pyridine-based **48j** resulted in a low yield. Noteworthy, the PKR of chiral enynes **48** led to a bridgehead quaternary stereocenter containing a C–F bond in a single step. Besides the intrinsic difficulty in generating quaternary stereocenters, the goal achieved is even more significant given the attention that the asymmetric introduction of fluorine at sp^3^ carbon centers has received in recent years [62–63]. A gram-scale synthesis was also successfully performed in five steps starting from the corresponding aldehyde in a 41% global yield.

**Scheme 26:**
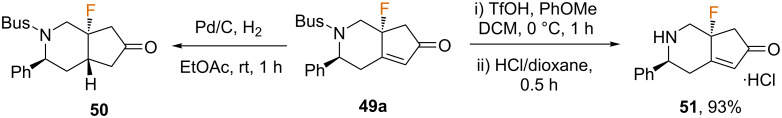
Examples of further modifications to the Pauson−Khand adducts [61].

Bicyclic product **49a** underwent diastereoselective (dr > 20:1) hydrogenation using palladium over activated charcoal under an atmosphere of hydrogen to afford saturated derivative **50** (Scheme 26). On the other hand, the *tert*-butanesulfonyl group could be removed through treatment of **49a** with trifluoromethanesulfonic acid in the presence of anisole to form **51**.

**Scheme 27:**
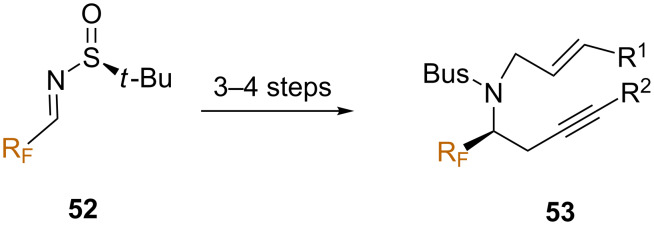
Asymmetric synthesis the fluorinated enynes **53**.

In a recent report, Fustero and co-workers synthesized a series of *N*-tethered 1,7-enynes **53** bearing fluorinated substituents starting from fluorinated *tert*-butanesulfinyl imines **52** (Scheme 27), which were later evaluated in the cobalt-mediated PKR [64].

**Scheme 28:**
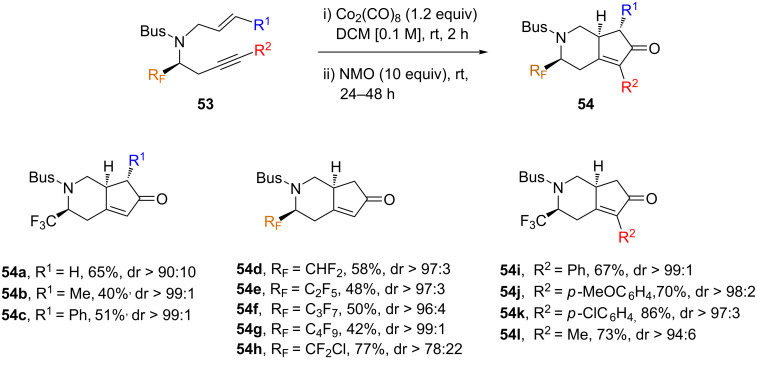
Intramolecular PKR of chiral *N*-tethered 1,7-enynes **53** [64].

The chiral enynes were treated with a stoichiometric amount of Co_2_(CO)_8_ in CH_2_Cl_2_ affording the corresponding cobalt complexes that, upon addition of an excess of NMO, underwent an efficient intramolecular PKR to afford the corresponding bicyclic cyclopentenones **54** as single diastereoisomers (Scheme 28). In general, yields were moderate to good, and high diastereoselectivities were observed in almost all cases.

**Scheme 29:**
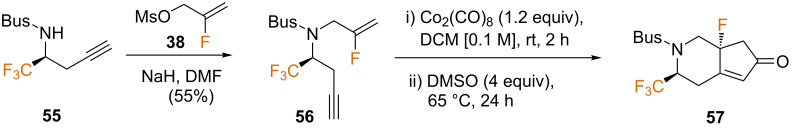
Intramolecular PKR of chiral *N*-tethered 1,7-enyne bearing a vinyl fluoride [64].

The PKR tolerated both mono- and disubstituted olefins well, but when a trisubstituted olefin was assayed in the PKR, the initial enyne was recovered, in agreement with previous precedents describing that trisubstituted alkenes are very unreactive substrates in this kind of process (Scheme 28) [65]. The influence of the introduction a vinyl fluoride moiety on the PKR was also investigated (Scheme 29). The resulting cyclopentenones **57** were obtained in a lower yield (52%) but high diastereoselectivity, similar to those previously reported [59,61].

**Scheme 30:**
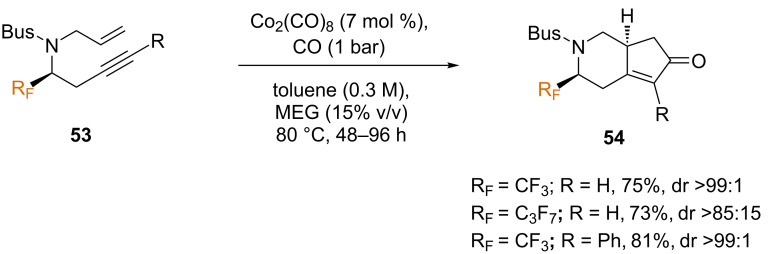
Catalytic intramolecular PKR of chiral *N*-tethered 1,7-enynes [64].

The authors also explored the PKR in a catalytic version based on a biphasic system of ethylene glycol/toluene, which generally enhanced both yields and stereoselectivities, as well as simplifying purification of the products [66]. This methodology (7 mol % catalyst, atmospheric CO pressure, and 15% v/v of ethylene glycol in toluene) was shown to be suitable for substrates bearing differing fluorinated groups, as well as one example with a phenyl-substituted alkyne, giving rise to products **54** in good yields and diastereoselectivities (Scheme 30).

### Intermolecular Pauson–Khand reactions of fluorine-containing compounds

**Scheme 31:**
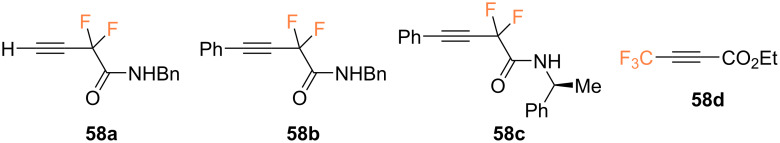
Model fluorinated alkynes used by Riera and Fustero [70].

In contrast to the previously discussed intramolecular PKR of fluorinated enynes, the study of intermolecular PKR reactions is far scarcer, and is limited by the poor reactivity and selectivity of simple alkenes. In this regard, most examples have been restricted to the use of ethylene or strained alkenes such as cyclopropene, norbornene, norbornadiene, (*E*)-cyclooctene, or bicyclo[3.2.0]hept-6-ene [67–69]. The first example of an intermolecular version of fluorinated compounds was reported by Riera, Fustero and co-workers in 2010 [70]. In this seminal study, four model fluorinated alkyne precursors **58** were synthesized (Scheme 31).

**Scheme 32:**
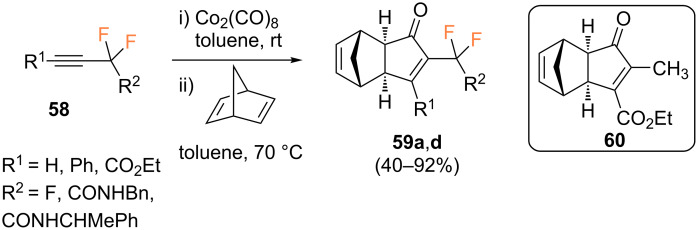
PKR with norbornadiene and fluorinated alkynes **58** [71].

With this small family of fluorinated alkynes, the authors studied the PKR using norbonadiene as the olefin partner under thermal conditions using a stoichiometric amount of Co_2_(CO)_8_. Products **59** were obtained in moderate to excellent yields, as single regioisomers, and **59c** as a 1:1 mixture of diastereoisomers. The most striking feature was the unexpected regiochemical outcome of this study; the fluorinated moiety occupied the α-position in the final cyclopentenone ring in all cases (Scheme 32). This was expected for terminal alkyne **58a**, since this is the substitution pattern always found (see Scheme 4). On the other hand, for alkynes bearing substituents of similar steric bulk, the electron withdrawing group is expected to occupy the β-position. However, for alkynes **58b,c** the opposite regiochemistry was found. Finally, the use of alkyne **58d** with two electron-withdrawing groups resulted in the regioselective formation of product **59d** in excellent yield, indicating an inherent trend of the fluoroalkyl group to occupy the α-position regardless of the steric or electronic nature of the other substituent. These results contrast with those obtained for non-fluorinated analogue (ethyl 2-butynoate) for which the expected regioisomer **60** was formed, bearing the methyl group at the α-position and the electron-withdrawing ester group in the β-position. These results may suggest that fluoroalkyl groups behave as bulky substituents rather than as electron-withdrawing ones, perhaps due to the purely inductive nature of the latter [71].

**Scheme 33:**
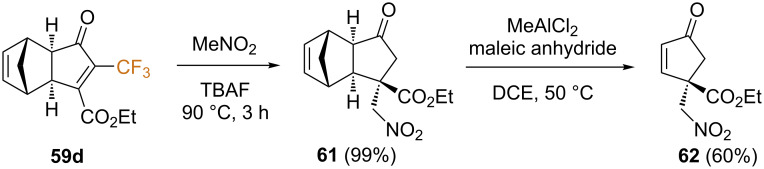
Nucleophilic addition/detrifluoromethylation and retro Diels-Alder reactions [70].

In the same work, the authors described the conjugated addition of several nucleophiles to model substrate **59d** (Scheme 33). In this sense, while the addition of hard organometallic nucleophiles, such as lithium dialkylcuprates or Grignard reagents, failed, softer nucleophiles such as nitroalkanes cleanly added to the β-position, providing the Michael adduct **61**. Unexpectedly, the conjugate addition reaction resulted in concomitant detrifluoromethylation. Furthermore, the Lewis acid-mediated retro Diels–Alder reaction was carried out uneventfully on product **61**, affording the corresponding cyclopentenone **62** in moderate yield (Scheme 33).

**Scheme 34:**
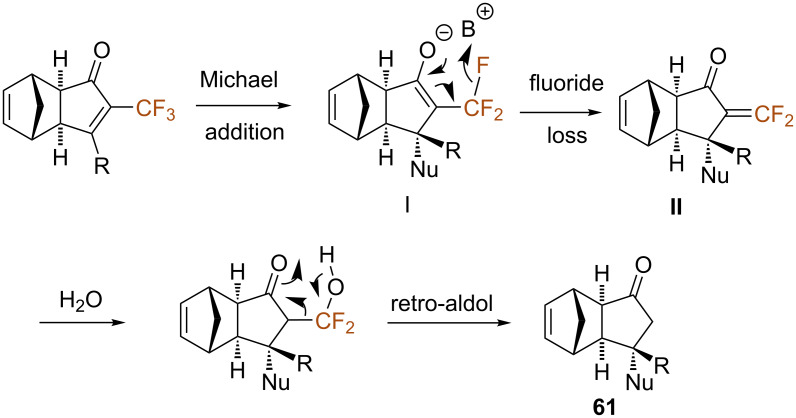
Tentative mechanism for the nucleophilic addition/retro-aldol reaction sequence.

In order to rationalize the unexpected loss of the trifluoromethyl group upon nucleophilic addition, the authors suggested the tentative mechanism depicted in Scheme 34. As shown, γ-fluoride loss from enolate **I**, formed after the conjugate Michael addition, would lead to the formation of difluoroenone **II**. This could in turn undergo a nucleophilic addition of water, followed by a retro-aldol reaction affording the final product (Scheme 34). This mechanism was experimentally supported by the beneficial effect of water and the observation of HF-loss by ^19^F NMR.

**Scheme 35:**
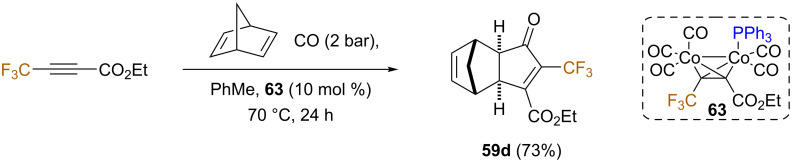
Catalytic PKR with norbornadiene [70].

The catalytic version of this process was also studied. The best results in terms of efficiency and stereoselectivity were obtained using the less reactive triphenylphosphine dicobaltpentacarbonyl complex **63** as the catalyst (Scheme 35).

**Scheme 36:**
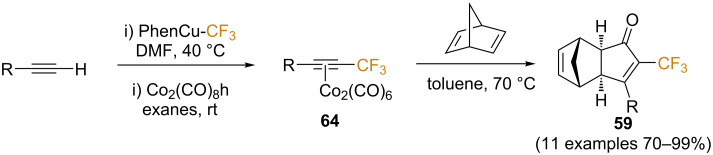
Scope of the PKR of trifluoromethylalkynes with norbornadiene [72].

In a later study [72], Riera and Fustero generalized the use of trifluoromethylalkynes as substrates for the PKR. The copper-catalyzed trifluoromethylation of terminal alkynes described by Qing and co-workers [73] allowed the efficient preparation of a small library of substrates bearing aryl, alkyl, and alkenyl substitutents. These were isolated after complexation to Co_2_(CO)_8_ as the corresponding adducts **64**, due to difficulties in their isolation. Subsequent heating with norbornadiene afforded the corresponding products **59** in good to excellent yields (Scheme 36).

**Scheme 37:**
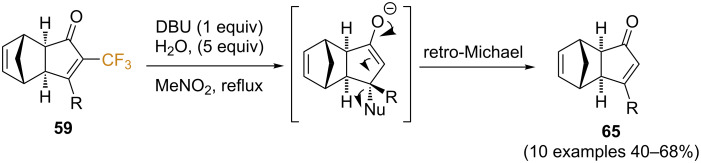
DBU-mediated detrifluoromethylation [72].

The authors then studied the elimination of the trifluoromethyl group from this library of PK adducts, building upon their own experience in the field (vide supra, Scheme 34). Thus, by subjecting enones **59** to treatment with DBU in wet nitromethane under reflux, clean conjugate addition/detrifluoromethylation was observed, in this case followed by retro-Michael reaction of nitromethane achieving enones **65** in moderate to good yields (Scheme 37). Interestingly, the overall reaction sequence results in the formal inversion of the regiochemistry for terminal alkynes, affording the products substituted at the β-position. This regiochemistry switch might be regarded as the Holy Grail in Pauson–Khand chemistry.

**Scheme 38:**
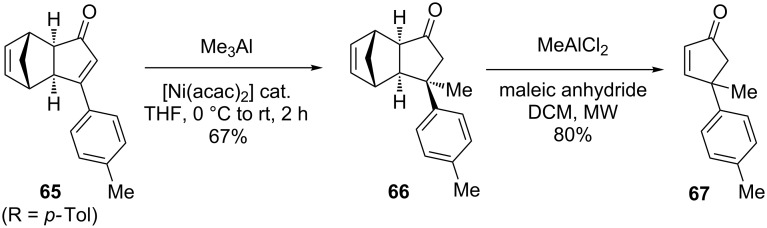
A simple route to enone **67**, a common intermediate in the total synthesis of α-cuparenone.

The synthetic potential of this methodology was demonstrated by the formal total synthesis of α-cuparenone [74–75], a bicyclic sesquiterpene that belongs to the cuparene family isolated from *Thuja orientalis* [76]. More specifically, the authors designed a simple route to enone **67**, a key intermediate in several previous total syntheses [77–78]. Its synthesis was achieved in two steps, namely a nickel-catalyzed conjugated addition of trimethylaluminum to form **66**, followed by Lewis acid-mediated retro Diels–Alder reaction (Scheme 38).

**Scheme 39:**
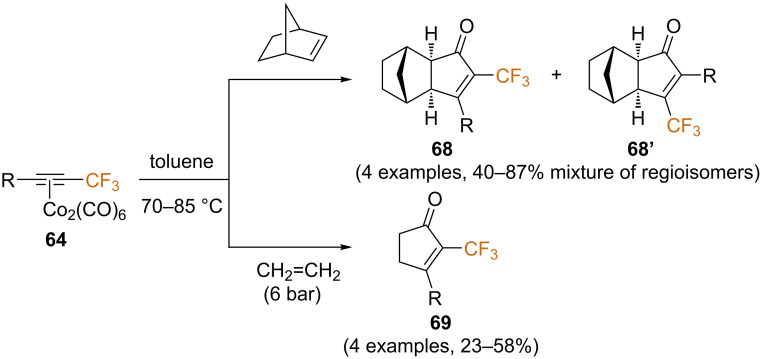
Effect of the olefin partner in the regioselectivity of the PKR with trifluoromethyl alkynes [79].

In another study by Riera and co-workers, they studied the influence of the olefin counterpart on the regioselectivity of the reaction [79]. Two olefins other than norbornadiene were used in this study, namely norbornene and ethylene (Scheme 39). Contrary to the results observed with norbonadiene and ethylene, which both furnished a single regioisomer, the use of norbornene afforded mixtures of regioisomers, although the same α-CF_3_ isomer was favored in all cases. The aforementioned DBU-mediated detrifluoromethylation (Scheme 37) was achieved for substrates **68** in most cases with low to moderate yields, while the corresponding products could not be isolated starting from substrates **69**.

**Scheme 40:**
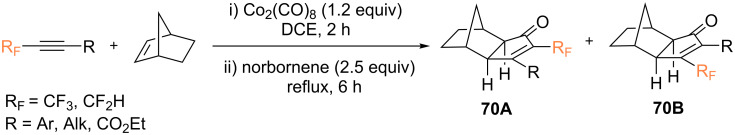
Intermolecular PKR of trifluoromethylalkynes with 2-norbornene reported by the group of Konno [54].

In 2012, Konno and co-workers reported the synthesis of 2-fluoralkyl-2-cyclopentenones through an intermolecular Co-mediated PKR of various trifluoromethyl alkynes with 2-norbornene (Scheme 40) [54]. In this process, the authors did not use NMO as a promoter due to its negative effect on the reaction yield. Under the described conditions, the cyclized products **70** were obtained as regioisomeric mixtures in moderate to high yields. The influence of alkyne substitution was studied, and alkynes bearing an electron-withdrawing or electron-donating group on the benzene ring afforded the corresponding cyclopentenones in high yields but as regioisomeric mixtures (ratio **A**/**B** ca. 70:30). Interestingly, a notable improvement of the regioselectivity was observed for trifluoromethyl alkynes bearing an alkyl substituent (R = Alk) or an ethoxycarbonyl group (R= CO_2_Et). When a difluoromethylated alkyne (R_F_ = CF_2_H) was used, the reaction took place very smoothly to preferentially afford the cyclopentenone with opposite regioselectivity (ratio **A**/**B** 31:69) to that observed for the other examples. This result contrasts with the lack of reactivity observed for CF_2_H-substituted enynes in the intramolecular version (see Scheme 15). Although the PKR was also investigated for other alkenes such as cyclohexene, maleic anhydride, ethylene carbonate, and 1-octene, the desired products were not formed under the reaction conditions.

**Scheme 41:**
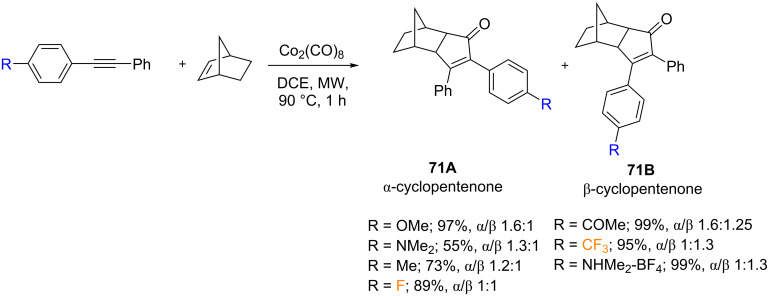
Intermolecular PKR of diarylalkynes with 2-norbornene reported by the group of Helaja [80].

In the same year, Helaja and co-workers examined the electronic effects of the alkyne substituent on the regioselectivity of the microwave-assisted PKR with norbornene [80]. The electronic effects were evaluated by altering one functional group in the *para*-position of the starting diarylalkynes (Scheme 41). In this regard, electron-donating substituents such as, methoxy, dimethylamine, and methyl favored the α-position in the final cycloadduct (**71A**), whereas electron-withdrawing substituents such as dimethylaminium, trifluoromethyl, and acetyl favored the β-regioisomer (**71B**). The 4-fluorine substituted diarylalkynes had a very weak EWG effect yielding an equimolar mixture of both regioisomers. The experimental results were confirmed by a DFT study of the NBO charges of the α-alkyne carbons, which also showed a correlation with the regioselectivity.

**Scheme 42:**
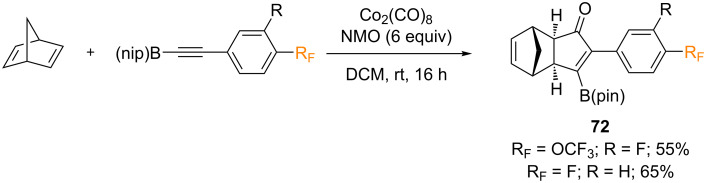
Intermolecular PKR reported by León and Fernández [81].

In 2016, León and Fernández reported the first intermolecular PKR of internal alkynylboronic esters with norbornadiene [81]. For this purpose, terminal alkynes were converted into their corresponding alkynylboronic pinacol esters, and evaluated in the intermolecular PKR with norbornadiene. Under the optimal reaction conditions, Co_2_(CO)_8_ (1 equiv), norbornadiene (3 equiv) and NMO (6 equiv) in dichloromethane, the internal alkynes afforded the corresponding α,β-substituted cyclopentenones with total stereo- and regioselectivity; the *exo*-stereoisomer with the B(pin) substituent in the β-position was obtained exclusively. The PKR was compatible with aromatic groups containing substituents with differing electronic properties, heteroaryl groups, benzopinacol, 1,8-diaminonaphthalene, olefinic, and aliphatic groups. However, the PKR failed when hindered alkynes were used. The authors reported two examples of fluorine-containing alkynylboronic pinacol esters, which afforded the corresponding β-substituted cyclopentenones **72** in good yields (Scheme 42).

**Scheme 43:**
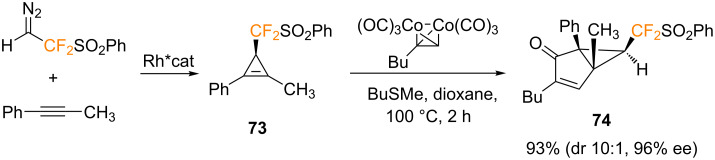
PKR reported with cyclopropene **73** [82].

In a recent report, Marek, Zhang, Ma and co-workers described a Rh^II^-catalyzed cyclopropenation reaction of internal alkynes with a difluorodiazoethane reagent, offering efficient access to a broad range of enantioenriched difluoromethylated cyclopropenes with almost quantitative yields and up to 97% ee [82]. This asymmetric carbene transfer reaction was performed using chiral Rh^II^ complexes, more specifically the Hashimoto catalyst ([Rh_2_(*S*-TCPTTL)_4_]) was found to be the most efficient in terms of yield and enantioselectivity. In this study, the authors described an example of an intermolecular PKR using the strained difluoromethylated cyclopropene **73** which, upon reaction with the hexacarbonyldicobalt complex prepared by treatment of the 1-butyne with Co_2_(CO)_8_, afforded the corresponding bicyclic cyclopropane-fused cyclopentenone **74** in high yield and high enantioselectivity (Scheme 43).

## Conclusion

In conclusion, as highlighted in this review, the PKR is still a hot area of chemical research as it provides access to cyclopentenones from accessible starting materials under relatively mild conditions. The increasing demand of fine chemicals bearing fluorine atoms at strategic positions, together with the ubiquitous presence of the cyclopentenone ring in added-value compounds, has attracted researchers’ interest throughout the past decades. Since its discovery, numerous research groups have devoted their efforts to develop alternative ways to expand the scope of the PKR and the combination of fluorinated building blocks with this [2 + 2 + 1] cycloaddition has been efficiently applied in the preparation of fluorinated compounds of biological interest. The addition of fluorine-containing olefins and alkynes to the arsenal of substrates for the PKR has resulted in a major contribution in the field. Not only has the scope of accessible products been significantly expanded, but a better understanding of the factors governing the regioselectivity has been achieved, including the possibility to formally reverse the regiochemistry in some cases. Despite the important advances highlighted in this review, there remains room for improvement in the scope of this useful reaction with fluorinated compounds. Nowadays, most described protocols for the fluorinated PKR are based on the cobalt-catalyzed version, and only a few examples have used other transition metal complexes. In this regard, significant advances can come from the careful selection of the metal complex and the CO source. The encouraging results described in this review will definitely pave the way for future applications in the fluoro-PKR, and put this emerging reaction at the forefront of drug design.
